# Real-time PCR assay to detect the novel Clade Ib monkeypox virus, September 2023 to May 2024

**DOI:** 10.2807/1560-7917.ES.2024.29.32.2400486

**Published:** 2024-08-08

**Authors:** Leonard Schuele, Leandre Murhula Masirika, Jean Claude Udahemuka, Freddy Belesi Siangoli, Justin Bengehya Mbiribindi, Pacifique Ndishimye, Frank M Aarestrup, Marion Koopmans, Bas B Oude Munnink, Richard Molenkamp, GREATLIFE MPOX group, Collaborators, Marjan Boter, Hayley Cassidy, Léandre Mutimbwa Mambo, Gitta Overbeek, Jean Pierre Musabyimana, Jules Ndoli, Saria Otani

**Affiliations:** 1 Department of Viroscience, Erasmus University Medical Center, Rotterdam, the Netherlands; 2 Centre de Recherche en Sciences Naturelles de Lwiro, South Kivu, DS Bukavu, Democratic Republic of the Congo; 3 SaBio Instituto de Investigación en Recursos Cinegéticos IREC (Universidad de Castilla-La Mancha & CSIC), Ciudad Real, Spain; 4 Congo Outbreaks, Research for Development, South Kivu, Bukavu, Democratic Republic of the Congo; 5 Department of Veterinary Medicine, University of Rwanda, Nyagatare, Rwanda; 6 Genomics Research and Development Division, Stansile Research Organization, Kigali, Rwanda; 7 Division Provinciale de la Santé, South Kivu, Bukavu, Democratic Republic of the Congo; 8 Research and Innovation Centre, African Institute for Mathematical Sciences (AIMS), Kigali, Rwanda; 9 Research Group for Genomic Epidemiology, National Food Institute, Technical University of Denmark, Kgs. Lyngby, Denmark; 10 The members of the network are listed under Collaborators; * These authors contributed equally to this work and share first authorship

**Keywords:** Monkeypox virus, Monkeypox virus Clade Ib, Mpox virus Clade Ib, Real-time PCR, diagnostic test

## Abstract

Monkeypox virus (MPXV) is an emerging zoonotic pathogen with complex epidemiology necessitating rapid diagnosis and distinguishing between clades and subclades. The emerging Clade Ib lacks the genomic region used in the Clade I-specific assay from the Centers for Disease Control and Prevention. We report an MPXV real-time PCR to specifically detect Clade Ib. The assay demonstrated proficient sensitivity and specificity in 92 samples and can be included along other TaqMan-based assays to detect MPXV and distinguish between clades and subclades.

In 2022, a global outbreak of Clade II monkeypox virus (MPXV) spread to more than 111 countries that had not previously reported cases, predominantly affecting the community of men who have sex with men in Europe and the Americas [1]. In the following year, the number of MPXV Clade I virus cases surged in Africa, with reports of more than 20,000 cases and 1,000 deaths spanning 25 of the 26 provinces in the Democratic Republic of the Congo (DRC) by June 2024 [2]. We recently showed that part of this is due to a separate mpox outbreak, which started in September 2023 in the South Kivu province from mostly heterosexual transmission and caused by a highly divergent Clade I virus, now designated Clade Ib [3]. However, genomic analysis of the newly detected Clade Ib genome revealed a deletion of the target sequence (within the C3L gene) currently used for identifying Clade I viruses [3-5]. This deletion, depending on the assay used, will impact the accuracy of real-time MPXV clade differentiation, leading to false negative identification of the novel Clade Ib strain. This may have important implications for monitoring the spread and control of the Clade Ib virus, especially in the context of the currently circulating Clade IIb MPXV which causes a milder form of disease. In this report, we describe the laboratory validation and subsequent implementation of a new real-time PCR assay to specifically detect Clade Ib. 

## Real-time PCR validation and implementation

The high sequence similarity between Clade I and Clade II MPXV (> 99%), as well as with other Orthopoxviruses (> 90%), renders it challenging to develop robust and clade-specific target sequences. Therefore, insertions and deletions in the genome are often used as target regions to discriminate between the different lineages [4,6].

We designed a specific probe sequence to anneal directly upstream and downstream of the deletion in Clade Ib; an illustration for this is appended in Supplementary Figure S1. An alignment of the sequence region up- and downstream of the deletion showed no match with Clade I or Clade II (Figure). In addition, a similar annealing temperature could be applied with other TaqMan real-time PCR assay targets. The new assay contains the probe with a 5’-reporter molecule (FAM) and a 3’-quencher molecule: (BHQ1) 5’-FAM-ATATTCAGGCGCATATCCACCCACGT-BHQ-3’, forward primer: 5’-AAGACTTCCAAACTTAATCACTCCT-3’ and reverse primer: 5’-CGTTTGATATAGGATGTGGACATTT-3’.

**Figure:**
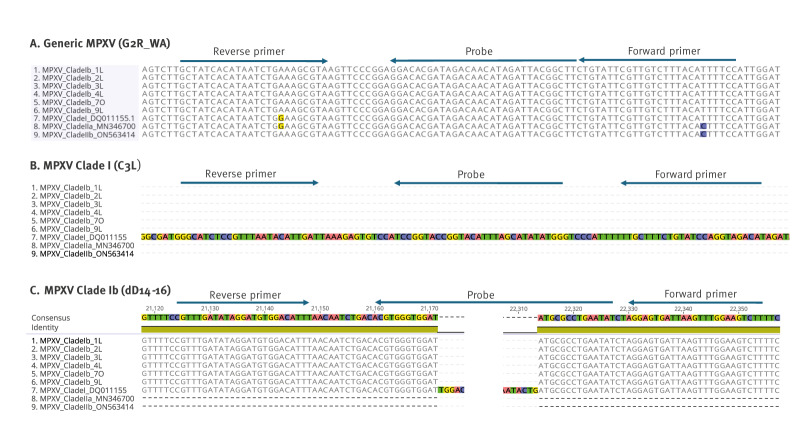
Alignment of primers and probes with monkeypox virus sequences, September 2023–May 2024

CDC: United States Centers for Disease Control and Prevention; MPXV: monkeypox virus.

Panel A: Generic MPXV primers and probes present with two mismatches in forward and reverse primers in Clade IIa and Clade IIb (highlighted in yellow and blue).

Panel B: Clade I CDC primers and probe are missing in Clade Ib and are only present in Clade I.

Panel C: Novel forward and reverse primers and probe spanning the region up and downstream of the deletion event in Clade Ib sequences. Deletion event of 1,140 bp compared with previously described Clade I sequences such as consensus sequence number 7 (MPXV_Clade1_DQ011155).

MPXV_CladeIb_1L/2L/3L/4L/7O/9L depict the first described Clade Ib sequences from Masirika et al. [3].

## Proof of principle and validation

High-performance liquid chromatography-purified primers and probes (Eurogentec) of our new Clade Ib assay (dD14-16), as well as the three MPXV assays recommended by the United States Centers for Disease Control and Prevention (CDC) for generic MPXV (G2R_G), Clade I (C3L) and Clade II (G2R_WA), all by Li et al. [4], were initially tested using synthesised DNA of the Clade Ib region (GeneArt), along with extracted Clade IIb DNA from stored patient samples from the 2022 outbreak in the Netherlands [7]. We applied the following PCR conditions: 400 nmol/L forward and reverse primers, 200 nmol/L TaqMan probe with 1 × TaqMan Universal PCR master mix (Thermo Fisher) and 8 μL of extracted or control MPXV DNA. The following thermal cycling conditions were applied: one cycle at 50 °C for 5 min, followed by 94 °C for 20 s, 45 cycles at 94 °C for 3 s and 60 °C for 30 s and run on a LightCycler480. Supplementary Figures S2, S3 and Table S1 provide additional insight into specificity testing, LOD estimation and refinement.

We determined the analytical sensitivity of the novel assay using the synthesised Clade Ib sequence diluted in 12 1:2 dilutions ranging from 320 to 0 copies/reaction. All concentrations were tested sixfold. The LOD at 95% confidence was determined at 6.6 copies/genome (95% confidence interval (CI): 5.1–13.0) by PROBIT analysis using SPSS statistics (IBM); for the LOD refinement we refer to the appended Supplementary Figure S3. The analytical sensitivity of MPXV Clade I (C3L) and Clade II (G2R_WA) was 11.4 copies/reaction (95% CI: 9.1–19.3) and 59.9 copies/reaction (95% CI: 43.5–110.1), respectively. This was comparable with previous assays by Li et al. [4] with 3.5 copies/reaction (Clade I) and 8.3 copies/reaction (Clade II) respectively.

## Real-time PCR evaluation

Evaluation of the real-time PCR assay for Clade I viruses was performed in Rwanda, along with researchers from the DRC during a capacity building training for outbreak research. We isolated MPXV DNA from a total of 92 samples from 91 patients with clinically suspected MPXV infection (67 skin lesion samples and 25 oropharyngeal swabs) that had been collected during the mpox outbreak in the South Kivu region between September 2023 and May 2024. These suspected mpox cases were tested both with the novel real-time assay (dD14-16) and the CDC real-time PCR assays [4]. The real-time PCR assays were run on a CFX96 (BIO-RAD) and analysed using CFX Maestro v2.3 (BIO-RAD). 

The novel assay (dD14–16) determined 82 of 92 samples as MPXV-positive (all distinguished as Clade Ib), highlighting the robustness of the approach. The Clade Ib (dD14-16) assay had one additional positive result (sample 75), compared with the generic CDC MPXV assay. We did not detect any Clade I- or Clade IIb-positive samples. The new assay showed quantification cycle (Cq) values comparable to the generic MPXV assay (G2R_G); see Supplementary Figure S4 and Table S2. Samples below Cq 30 were selected for MPXV whole genome sequencing (WGS) and later confirmed as Clade Ib [8]. As anticipated by Masirika et al. [3], the CDC-recommended Clade I assay (C3L) did not detect the novel Clade Ib (Table).

**Table:** Results of the monkeypox virus Clade Ib real-time PCR assay (dD14-16), South Kivu, Democratic Republic of the Congo, September 2023–May 2024 (n = 92)

Sample number	MPXV real-time PCR targets and respective Cq values			
	CDC MPXV (G2R_G)	CDC Clade I (C3L)	Clade Ib (dD14–16)	CDC Clade II (G2R_WA)
**Skin lesion**				
1	31.99	ND	32.01	ND
2	31.44	ND	32.18	ND
3	22.81	ND	22.97	ND
4	30.82	ND	31.28	ND
5	26.28	ND	26.29	ND
6	20.52	ND	20.39	ND
7	14.35	ND	14.62	ND
8	13.5	ND	13.4	ND
9	ND	ND	ND	ND
10	30.65	ND	30.58	ND
14	14.53	ND	14.65	ND
15	20.18	ND	21.07	ND
16	10.81	ND	13.16	ND
17	11.07	ND	11.64	ND
18	34.69	ND	34.6	ND
19	17.72	ND	18.25	ND
20	22.37	ND	23.63	ND
21	28.47	ND	28.5	ND
22	13.86	ND	13.72	ND
23	16.43	ND	16.46	ND
24	19.21	ND	19.63	ND
25	11.82	ND	13.09	ND
26	16.25	ND	17.03	ND
27^a^	18.25	ND	17.78	ND
28	17.97	ND	17.23	ND
29	18.59	ND	18.78	ND
30	19	ND	18.91	ND
31	13.61	ND	14.35	ND
32	31.38	ND	31.55	ND
33	15.83	ND	15.64	ND
34	16.91	ND	16.78	ND
35	23.73	ND	23.97	ND
36	15.68	ND	15.48	ND
37	15.02	ND	15.48	ND
38	14.87	ND	14.95	ND
39	22.41	ND	18.7	ND
40	12.98	ND	13.28	ND
41	13.44	ND	13.57	ND
42	20.18	ND	20.09	ND
43	18.71	ND	18.93	ND
44	18.46	ND	18.7	ND
46	12.24	ND	11.53	ND
47	31.6	ND	31.98	ND
48	10.96	ND	9.56	ND
52	21.4	ND	20.94	ND
53	15.39	ND	15.29	ND
59	15.13	ND	15.35	ND
60	18.88	ND	18.32	ND
61	25.03	ND	24.06	ND
63	17.15	ND	17.98	ND
64	34.17	ND	34.51	ND
65	30.07	ND	30.36	ND
66	17.77	ND	17.71	ND
67	ND	ND	ND	ND
70	21.78	ND	22.39	ND
71	ND	ND	ND	ND
73	24.85	ND	25.37	ND
74	18.41	ND	18.29	ND
75	ND	ND	25.39	ND
76	21.75	ND	21.4	ND
79	15.84	ND	15.93	ND
80	20.28	ND	20.33	ND
82	15.41	ND	13.61	ND
84	16.46	ND	16.23	ND
86	21.57	ND	22.36	ND
88	21.12	ND	21.44	ND
89	25.07	ND	25.54	ND
**Oropharyngeal swab**				
11	36.12	ND	35.6	ND
12	ND	ND	ND	ND
13	32.54	ND	34.12	ND
45	21.2	ND	20.61	ND
49	ND	ND	ND	ND
50	35.68	ND	35.84	ND
51	20.23	ND	20.44	ND
54	35.27	ND	36.71	ND
55	20.99	ND	20.27	ND
56	32.09	ND	31.96	ND
57	24.04	ND	23.4	ND
58	32.22	ND	10.44	ND
62^a^	21.17	ND	20.93	ND
68	ND	ND	ND	ND
69	ND	ND	ND	ND
72	26.98	ND	26.99	ND
77	33.01	ND	33.15	ND
78	18.31	ND	17.6	ND
81	21.79	ND	20.91	ND
83	34.39	ND	35.29	ND
85	ND	ND	ND	ND
87	ND	ND	ND	ND
90	34.19	ND	35.13	ND
91	33.55	ND	34.67	ND
92	16.03	ND	15.02	ND
**Negative control**	ND	ND	ND	ND

CDC: United States Centers of Disease Control and Prevention; Cq: quantification cycle; C3L: Clade I CDC assay; dD14–16: Clade Ib assay (this study); G2R_G: generic MPXV CDC assay; G2R_WA: Clade II CDC assay; MPXV: monkeypox virus; ND: not detected.

^a^ Samples 27 and 62 were collected from the same patient.

## Discussion 

In recent decades, human MPXV infections were largely linked to zoonotic spillover events with short estimated human-to-human transmission chains, mainly within or linked to endemic countries [9-11]. Monkeypox virus forms two distinct genomic clades: Clade I (formerly Congo Basin strain), which is prevalent in Central Africa, and Clade II (formerly West African strain), which is historically enzootic in West Africa. Although considerably higher case fatality rates have been reported for Clade I, robust estimates are lacking due to limited standardised studies. 

Real-time PCR is a fast, economical and sensitive assay to detect, distinguish and monitor MPXV outbreaks [4,12-14]. A few real-time PCR assays recommended by the CDC have been proposed, which distinguish MPXV from other orthopoxviruses, but also between Clade I and Clade II [4]. Alternative Clade I/II differential MPXV assays have been reported which target alternative regions to C3L gene [15]. However, it is unclear if they could differentiate between Clade I and Clade Ib. The CDC assay is most commonly applied and widespread. Our real-time PCR can be a useful addition to efficiently and economically screen for possible Clade Ib multicountry transmission or travel-related spread, including within Europe.

We developed, validated and implemented a novel real-time PCR assay (dD14-16) to detect the new MPXV Clade Ib. The assay can be performed alongside other TaqMan-based real-time targets to detect MPXV and distinguish between Clade I and Clade II. We were able to evaluate our dD14-16 real-time PCR assay for suspected mpox cases in Rwanda and could confirm all cases positive in the generic CDC MPXV assay were Clade Ib. As a result, this new assay can directly impact MPXV clade differentiation and monitoring. 

A limitation of this study was our inability to perform the assay in duplicate or reproduce results due to insufficient remaining MPXV DNA. Another limitation was the unavailability of MPXV Clade I DNA (non-Clade Ib) or of other orthopoxviruses for specificity testing of the new assay. However, by testing a total of 92 samples across two different sample matrices and confirming the presence of Clade Ib in 63 isolates (with a Cq value below 30) using WGS highlights the robustness of the new assay. Future investigations could include multiplexing all targets within one real-time assay to improve efficiency, along with comparing assay performance in additional sample materials. By quickly assessing mpox prevalence in humans, as well as animals, we can begin to understand the recent outbreak within eastern DRC.

## Conclusion

Following identification of a novel sublineage of Clade I MPXV in South Kivu (Clade Ib), and the observation of a deletion affecting commonly used typing assays, we developed and validated a new Clade Ib specific real-time PCR assay to allow rapid subclade assignment against the backdrop of other Clade I strains and globally circulating Clade IIb MPXV. This assay will help to understand the transmission pattern and geographical expansion of the newly identified Clade Ib outbreak. 

## Collaborators

Department of Viroscience, Erasmus University Medical Center, Rotterdam, Netherlands: Marjan Boter, Hayley Cassidy, Gitta Overbeek. Research Group for Genomic Epidemiology, National Food Institute, Technical University of Denmark, Kgs. Lyngby, Denmark: Saria Otani. Research Innovation and data Science Division, Rwanda Biomedical Center, Kigali, Rwanda: Jean Pierre Musabyimana. Research and Clinical Education Department, University Teaching Hospital of Butare, Huye, Rwanda: Jules Ndoli. Zone de Santé de Kamituga, Kamituga, South Kivu, Bukavu, Democratic Republic of the Congo: Léandre Mutimbwa Mambo.

## Supplementary Data

321000 Supplement
