# Authors’ correction for Euro Surveill. 2024;29(32)

**DOI:** 10.2807/1560-7917.ES.2024.29.36.240905c

**Published:** 2024-09-05

**Authors:** Eurosurveillance editorial team

**Affiliations:** 1 European Centre for Disease Prevention and Control (ECDC), Stockholm, Sweden

At the request of the authors, the following changes were made on 30 August 2024 in the article entitled ‘Real-time PCR assay to detect the novel Clade Ib monkeypox virus, September 2023 to May 2024’ by Schuele et al., originally published on 8 August 2024: MPXV (G2R-WA) in the heading of panel A was corrected to read MPXV (G2R). Figure 1A was replaced, highlighting only the primer-target mismatches. Reference to Rwanda was removed in one sentence in the Discussion.

