# Supplementary material for: Real-time PCR assay to detect the novel Clade Ib monkeypox virus, September 2023 to May 2024
Source: Euro Surveill. 2024 Aug 8;29(32):2400486. doi: 10.2807/1560-7917.ES.2024.29.32.2400486 (PMC11312019; doi:10.2807/1560-7917.ES.2024.29.32.2400486)
Supplement: Supplement [file 24-00486_SCHUELE_Supplement.pdf]

Supplementary Material

This supplementary material is hosted by *Eurosurveillance* as supporting information alongside the article “Real-time PCR assay to detect the novel Clade Ib monkeypox virus”, on behalf of the authors, who remain responsible for the accuracy and appropriateness of the content. The same standards for ethics, copyright, attributions and permissions as for the article apply. Supplements are not edited by *Eurosurveillance* and the journal is not responsible for the maintenance of any links or email addresses provided therein.

Initial proof of principle

The synthesised DNA from Clade Ib (5’ATTAGAATTTTCTATTTCAACGGGTATAGCAGAATATTTGAAACACGGCACTT
CGAAATGGAAAAGACTTCCAAACTTAATCACTCCTAGATATTCAGGCGCATATC
CACCCACGTGTCAGATTGTTAAATGTCCACATCCTATATCAAACGGAAAACTTCT
AGCGGCTTAAAAGATCATACACTCATACAACGACAATGTAGACTTTAAGTGC-3’), along with other MPXV control sequences were serially diluted (10-fold) and tested with the discrimination PCRs from Li et al. (2010) and the novel PCR assay. This gave an initial estimation of the LoD, which ranged from 6 to 233 copies/reaction (at 95% confidence). All negative controls (12 replicates per assay) were undetectable. Previously isolated MPXV Clade IIb DNA from patient samples in the recent global outbreak (2022) tested positive only in the Clade II-specific PCR. MPXV negative patient samples showed no detection in any PCRs (Table S1).

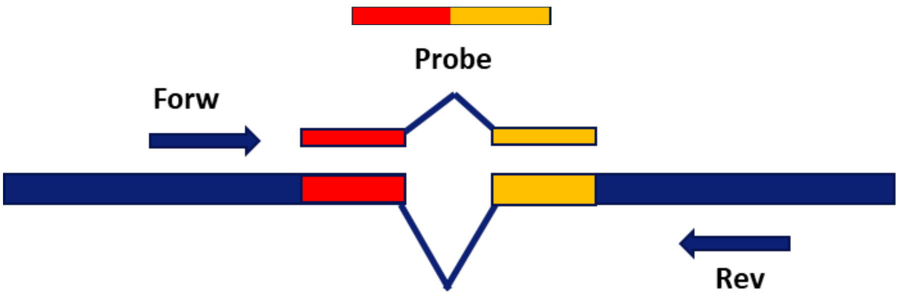

Figure S1. Illustration of the new primer probe target highlighting the probe design which spans up and downstream of the deleted region. **Abbreviations:** Forw, forward primer; Rev, reverse primer.

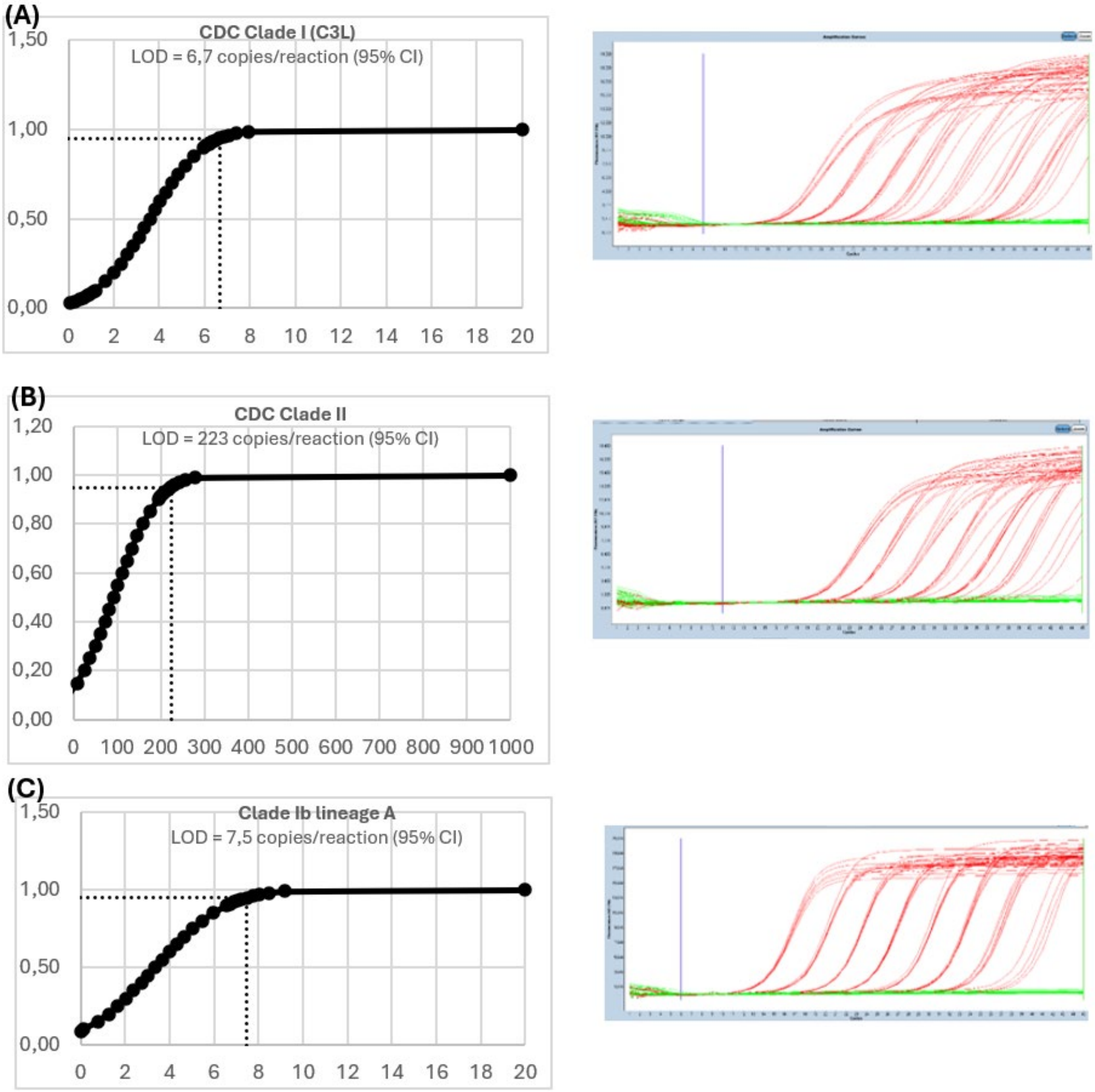

Figure S2. LoD estimation and amplification profiles of serial DNA dilutions with MPXV real-time PCR assay targets: (a) Clade I, (b) Clade II and (c) Clade Ib. Reaction mixtures contain: 0.4 µmol/L forward (5'-AAGACTTCCAAACTTAATCACTCCT-3') and reverse primer 5'-CGTTTGATATAGGATGTGGACATTT-3'; 200 nmol/L TaqMan probe (5'-FAM-ATATTCAGGCGCATATCCACCCACGT-BHQ-3') with 1x TaqMan Universal PCR Master Mix (Thermo Fisher) and 8 µL of extracted DNA. Thermal cycling conditions: One cycle 50°C for 5min, 95 °C for 20 s; 45 cycles 92°C for 3 sec, 60 °C for 30 sec.

Table S1. Specificity testing of the different MPXV typing PCRs. MPXV DNA from anonymized patient samples (Clade IIb) and MPXV negative samples were tested with generic (MPXV), Clade II, Clade I, and Clade Ib PCRs. Ct values are shown.

Sample #	CDC MPXV (G2R_G)	CDC Clade II (G2R_WA)	CDC Clade I (C3L)	Clade Ib (dD14-16)
1	23.6	25.0	ND	ND
2	ND	ND	ND	ND
3	20.6	21.7	ND	ND
4	27.7	29.3	ND	ND
5	31.7	33.5	ND	ND
6	ND	ND	ND	ND
7	ND	ND	ND	ND
8	23.3	24.5	ND	ND
9	ND	ND	ND	ND
10	39.7	39.5	ND	ND
11	ND	ND	ND	ND
12	34.3	37.0	ND	ND
13	ND	ND	ND	ND
14	ND	ND	ND	ND
15	ND	ND	ND	ND
16	ND	ND	ND	ND
17	ND	ND	ND	ND
18	25.0	26.5	ND	ND
19	ND	ND	ND	ND
20	ND	ND	ND	ND

Abbreviations: ND, not detected; Ct, cycle threshold

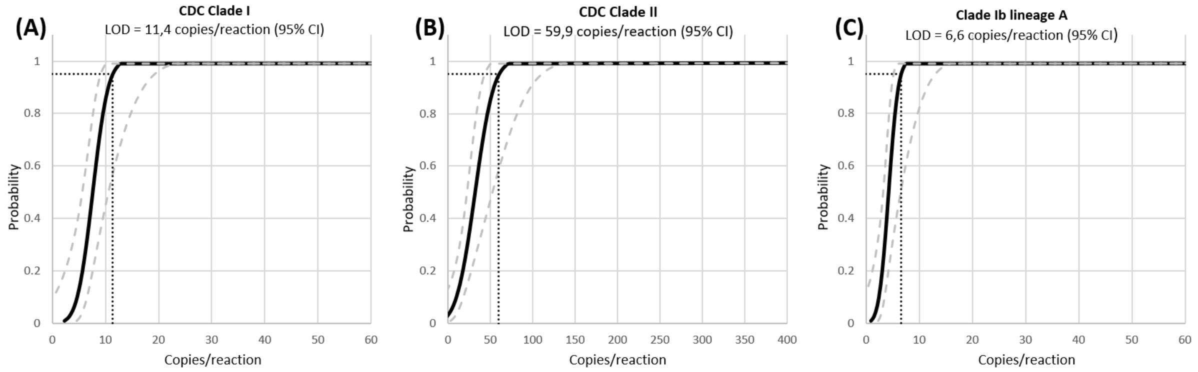

Figure S3. Determination of refined LOD of three qPCR assays. Black and grey lines highlight the 95% CI. **(a)** MPXV-Clade I: 11.4 copies/reaction (95% CI = 9.1 – 19.3), **(b)** MPXV-Clade II: 59.9 copies/reaction (95% CI = 43,5 – 110,1), **(c)** MPXV-clade-Ib: 6.6 copies/reaction (95% CI = 5.1 – 13.0).

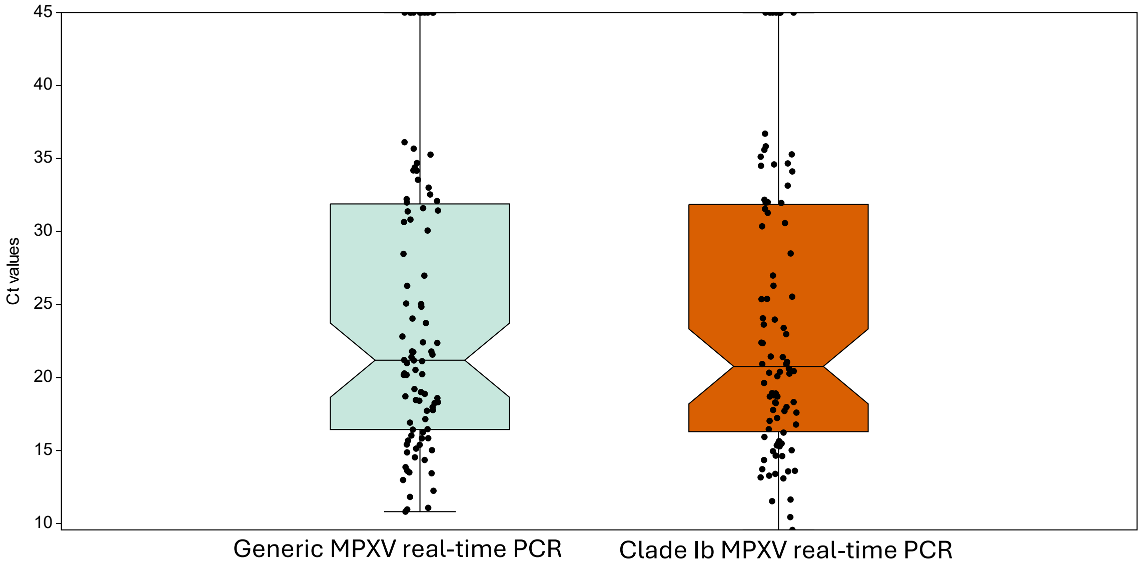

Figure S4. Notched box plot showing Ct values of generic CDC MPXV and novel Clade Ib real-time PCR

Table S2. Statistical comparison of Ct values between generic and Clade Ib MPXV real-time PCR

	Generic MPXV	*Clade Ib*
Mean	24,33608696	23,94858696
Variance	98,75415155	97,01915073
Observations	92	92
Pearson Correlation	0,949840696	
Hypothesised Mean Difference	0	
df	91	
t Stat	1,185636915	
P(T<=t) one-tail	0,119426228	
t Critical one-tail	1,661771155	
P(T<=t) two-tail	0,238852457	
t Critical two-tail	1,986377154	
